# Prediction of reduction potentials from calculated electron affinities for metal-salen compounds

**DOI:** 10.3762/bjoc.5.82

**Published:** 2009-12-23

**Authors:** Sarah B Bateni, Kellie R England, Anthony T Galatti, Handeep Kaur, Victor A Mendiola, Alexander R Mitchell, Michael H Vu, Benjamin F Gherman, James A Miranda

**Affiliations:** 1 Department of Chemistry, California State University, Sacramento, 6000 J Street, Sacramento, CA 95819-6057, United States

**Keywords:** density functional theory, electron affinity, metal-salen, reduction potential

## Abstract

The electron affinities (EAs) of a training set of 19 metal-salen compounds were calculated using density functional theory. Concurrently, the experimental reduction potentials for the training set were measured using cyclic voltammetry. The EAs and reduction potentials were found to be linearly correlated by metal. The reduction potentials of a test set of 14 different metal-salens were then measured and compared to the predicted reduction potentials based upon the training set correlation. The method was found to work well, with a mean unsigned error of 99 mV for the entire test set. This method could be used to predict the reduction potentials of a variety of metal-salen compounds, an important class of coordination compounds used in synthetic organic electrochemistry as electrocatalysts.

## Introduction

The electroreductive cyclization (ERC) reaction is a process in which an electron-deficient alkene that is tethered to an acceptor (e.g., an aldehyde or ketone) undergoes an electrochemically promoted reductive cyclization leading to the formation of a new sigma bond between the β-carbon of the alkene and the acceptor unit [1]. The ERC reaction has been applied towards the total synthesis of many complex natural products, including quadrone and phorbol [2–3].

Previously, it has been shown that Ni(II)-salen can serve as an electrochemical mediator in the ERC reaction [4]. The most significant advantage of this variant of the ERC reaction was the ability to effect the reaction at a more positive potential than the unmediated ERC reaction, resulting in a more chemoselective reaction [5]. The postulated mechanism of the reaction operated via a Ni(II)-salen radical anion as the active catalyst and evidence for the formation of a Ni(II)-salen radical anion during electrolysis was later put forth by Peters and co-workers [6].

In mediated ERC, while Ni(II)-salen (reduction potential or *E_pc_* = −2.1 V vs. Ag/AgNO_3_) is an effective electrochemical mediator, the analogous Co(II)-salen (*E_pc_* = −1.6 V vs. Ag/AgNO_3_) fails to promote cyclization. Direct ERC (unmediated) occurs at a reduction potential of −2.7 V vs. Ag/AgNO_3_. It was concluded that the 1.1 V thermodynamic barrier was too large to allow electron transfer to occur from the reduced form of the Co(II)-salen to the substrate [4].

We sought to discover if there were other metal-salen compounds that also fall within an “electrochemical potential window” in which effective ERC would occur. The electrochemistry of a few metal-salens is known in the literature; however, there are a great many possible metal-salens that we would like to investigate as electrocatalysts that have unknown electrochemistry.

Fry demonstrated that the density functional B3LYP/6-31G(d) level of theory can be used to accurately predict the reduction potentials for a series of chalcones [7]. The electron affinities (EAs) of a training set of 29 monosubstituted chalcones were computed, while the reduction potentials of the training set were measured experimentally. The EAs and reduction potentials of the training set showed a linear correlation (R^2^ = 0.969). The reduction potentials of an additional 72 di-, tri-, and tetrasubstituted chalcones were then accurately predicted from computed EAs using the linear correlation derived from the training set. In addition, Fry has also recently published an account of a similar procedure in the prediction of oxidation potentials of substituted triphenylamines using density functional theory [8]. In that work, Fry explored whether a potential substituted triphenylamine was likely to have a high enough oxidation potential to be useful as an electrocatalyst for a difficultly oxidized organic substrate before embarking upon a proposed synthesis of the substituted triphenylamine.

By using Fry’s method of calculating EAs to predict reduction potentials, we hoped to establish a method of assessing the suitability of prospective metal-salen catalysts other than Ni(II)-salen as electrocatalysts in the ERC reaction. The indirect computation of reduction potentials in this manner avoids the need to compute solvation enthalpies, eliminating the uncertainties associated with calculating solvation energies of ions [9]. A training set comprised of 19 metal-salen compounds, containing a variety of metal centers and electron-donating and electron-withdrawing substituents, was synthesized via a two-step process. The reduction potentials (*E_pc_*) of the 19 metal-salens were then measured experimentally using cyclic voltammetry. Analogous methods were applied to obtain the *E_pc_* for a test set of 14 metal-salens employing a variety of new metals and substituents. Concurrently, density functional theory calculations enabled computation of EAs [10] for both sets of metal-salens.

## Results and Discussion

**Figure 1:**
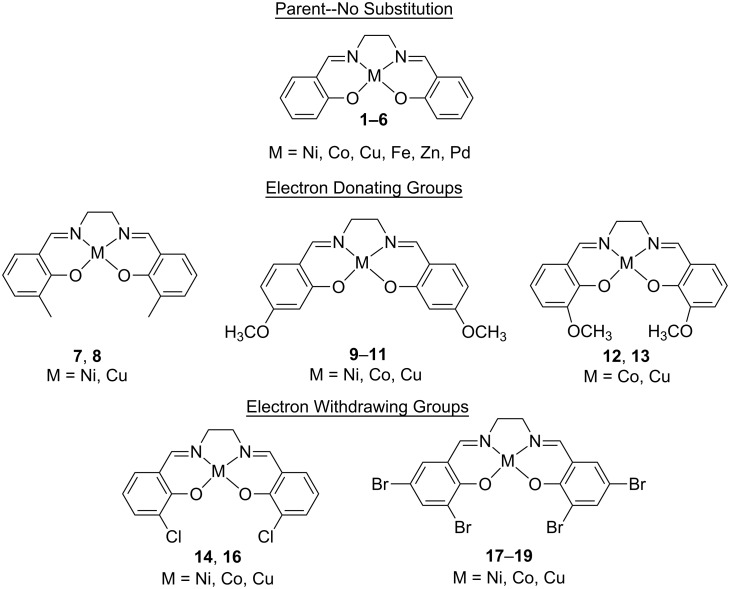
Training set of 19 metal-salens.

The 19 metal-salens comprising the training set are shown in Figure 1. The metals used in the training set include nickel, cobalt, copper, iron, zinc, and palladium. Included in the training set are the parent metal-salens (no substitution around the salen ligand), as well as metal-salens with various electron-donating and electron-withdrawing groups around the periphery of the salen ligand.

**Figure 2:**
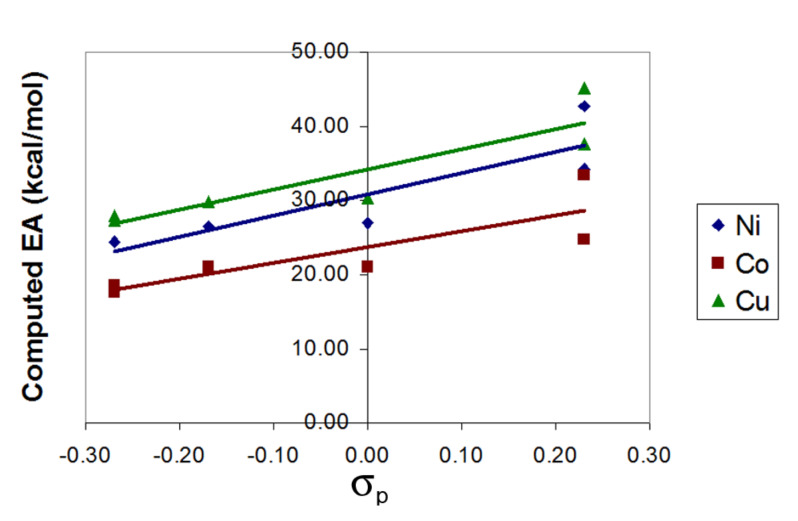
Correlation between electron affinity (EA) and Hammett σ_p_ parameter in the training set (R^2^ = 0.76, 0.72, and 0.81 for Ni, Co, and Cu, respectively).

Figure 2 shows that there is a good linear correlation between the computed EA and the Hammett parameter σ_p_ for the substituents [11]. Only nickel, cobalt, and copper were plotted on the graph due to the other metals (iron, zinc, and palladium) having only one point each in the training set. Negative Hammett parameters result from the presence of electron-donating groups and lead to smaller EAs. Positive Hammett parameters result from the presence of electron-withdrawing groups and lead to larger EAs.

**Figure 3:**
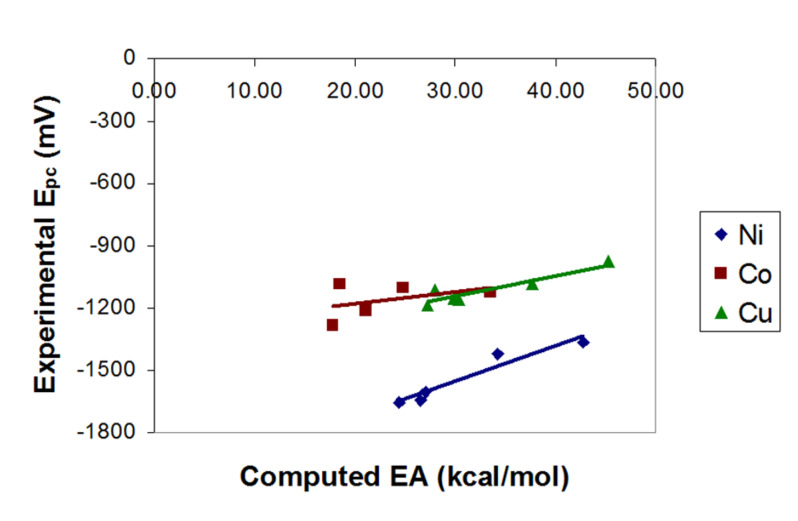
Correlation between *E_pc_* and EA for the Ni, Co, and Cu training set metal-salens (R^2^ = 0.93, 0.17 (0.63 excluding salen **10**), and 0.86 for Ni, Co, and Cu, respectively).

Figure 3 shows that there is a good linear correlation between the experimental reduction potential (*E_pc_*) and EA for each of nickel, cobalt, and copper. Iron, zinc, and palladium were again excluded due to having only one point each in the training set.

**Figure 4:**
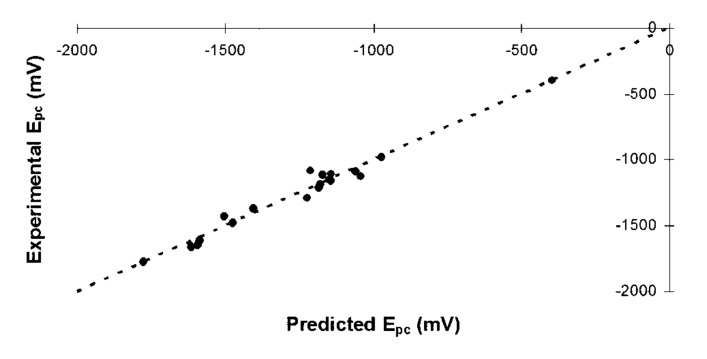
Comparison of experimental and predicted *E_pc_* for all training set metal-salens.

Finally, based upon the entire training set, the reduction potentials for the test set metal-salens can be predicted according to Equation 1 (where EA is in kcal/mol and *E_pc_* in mV). Once an EA has been calculated for a given metal-salen, the reduction potential can be calculated from the appropriate equation. Figure 4 shows the entire training set of 19 metal-salens, plotting experimental *E_pc_* versus calculated *E_pc_* based upon Equation 1. The plot is highly linearly correlated (R^2^ = 0.973). The slope of the correlation in Equation 1 (i.e., 11.406) is representative of the average value of the slopes if the EA is correlated to the *E_pc_* for each metal individually (cf. Figure 3, where the slopes are 17.194, 5.556, and 9.945 for Ni, Co, and Cu, respectively).

[1]
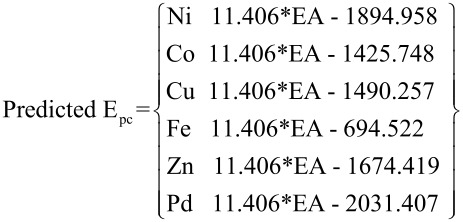


**Figure 5:**
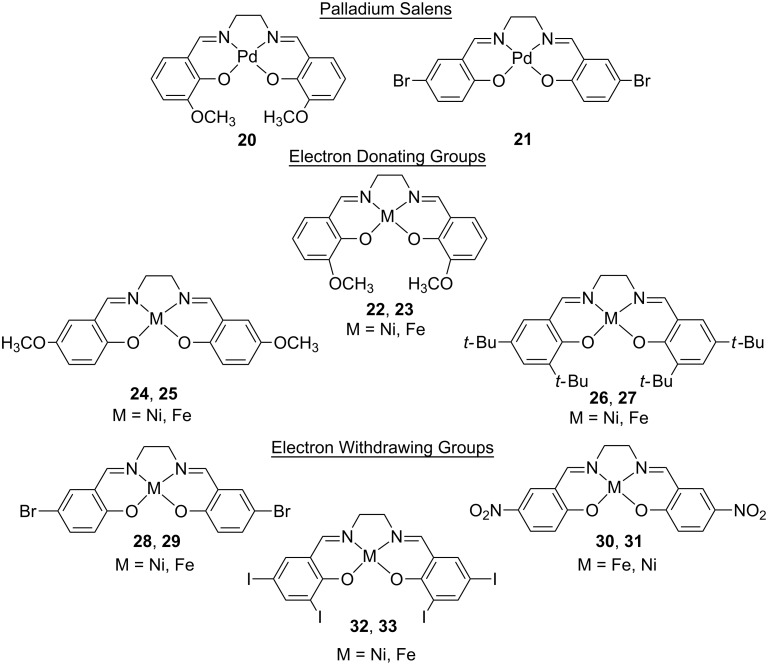
Test set of 14 metal-salens.

In order to test the effectiveness of the training set correlation in predicting the reduction potentials of new metal-salens, we used the correlation with computed EA values to predict the *E_pc_* for a test set comprised of new palladium, nickel, and iron metal-salens with various electron-withdrawing and electron-donating groups around the periphery of the salen ligand (Figure 5).

Mean signed and unsigned errors for the predicted versus experimental *E_pc_* are shown in Table 1. Overall, the test set had a mean signed error of −16 mV and a mean unsigned error of 99 mV. These results demonstrate that the training set correlation is transferable to the test set.

**Table 1:** Errors in the predicted *E_pc_* vs. experimental *E_pc_* values for the test set metal-salens.

	Pd-salens	Ni-salens	Fe-salens	entire test set

mean signed error (mV)	−111	66	−66	−16
mean unsigned error (mV)	111	116	78	99

## Conclusion

We have effectively used computational chemistry to predict the reduction potentials of metal-salen compounds. A training set of 19 metal-salen compounds was used to build a correlation between computed EAs and experimental reduction potentials. This correlation was used to predict the reduction potentials of a test set of 14 metal-salen compounds with a mean unsigned error of 99 mV. We are currently examining the data gathered in this study in order to find potential electrocatalysts in mediated ERC reactions. We are also undertaking an examination of the electronics and frontier orbitals from the calculations on the metal-salens to reveal the most likely site of reduction in each of the metal-salens. We are anticipating that this data would shed light upon the mechanism of mediated ERC reactions using reduced nickel(II)-salen. Results of these studies will be reported in due course.

## Experimental

*Computational Methods*: All computations were carried out using density functional theory methods in Gaussian03 [12]. In geometry optimizations, the 6-31G(d,p) basis set was used for all atoms, except the metal, for which the Stuttgart effective core potential basis set [13] was used. For subsequent single-point energy calculations, the 6-311++G(d,p) basis set was used for the non-metal atoms. Vibrational frequencies were computed for each optimized geometry in order to verify them as stationary points and to obtain zero-point energies and thermal enthalpy corrections, such that enthalpies at 25 °C could be obtained. The electron affinity (EA) for a given metal-salen was then the opposite of the enthalpy change for the 1-electron reduction reaction (Equation 2).

[2]
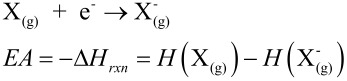


The choice of density functional to employ here was made from among the pure density functionals BLYP [14–15], OLYP [15–16], and HCTH [17] and the hybrid density functionals B3LYP [14–15,18], O3LYP [15–16,19] and B97-1 [17,20]. First, structures for unsubstituted and 4-hydroxy Ni(II)-salens were optimized with each of the six functionals and compared to crystal structures for these compounds determined by Kondo et al. [21]. Second, EAs were computed for Ni(II)-salen with the standard aliphatic bridging group and with an olefinic bridging group, for both of which experimental EAs are available [22]. With an average RMSD of 0.028 Å (for metal-ligand bond lengths only, 0.022 Å) for the optimized versus crystal structure geometries and an average ΔEA of 3.67 kcal/mol for computed versus experimental EA, B97-1 was determined to be the optimal functional for reproducing both key geometric and electronic features of the metal-salens.

Among the metal-salens considered in the training and test sets, only one possible spin state need be considered for the neutral and reduced forms of the Cu(II) (doublet and singlet, respectively) and Zn(II) (singlet and doublet, respectively) salens. For the complexes including Ni(II) and Pd(II), the ground state of the neutral form was in all cases found to be the singlet spin state, while only the doublet state is possible for the one-electron reduced forms. Multiple spin states exist for the Co(II)-salens (neutral: doublet and quartet; reduced: singlet and triplet) and Fe(II)-salens (neutral: singlet, triplet, and quintet; reduced: doublet and quartet). For all substituents, the ground states proved to be quartet and singlet for the neutral and reduced Co(II)-salens, and quintet [23] and doublet for the neutral and reduced Fe(II)-salens. All calculated EAs are based upon these lowest energy spin states.

*Synthesis of metal-salens: representative procedure*: The metal-salens were synthesized by a standard procedure [24]. First, the salen ligand was made by condensation of the appropriate substituted salicylaldehyde with ethylenediamine. The reaction was allowed to reflux for 2 h before the crude salen ligand was recrystallized in 95% ethanol. Each salen ligand was characterized by IR and NMR spectroscopy. Second, the appropriate metal acetate and the salen ligand were allowed to reflux for 1 h before the crude metal-salen was recrystallized in 95% ethanol. Each metal-salen was characterized by IR and High Resolution-Mass Spectrometry.

*Voltammetry*: Cyclic Voltammetry was carried out at room temperature at 100 mV s^−1^ at a glassy carbon electrode in spectral grade dimethylformamide/0.1 M Bu_4_N^+^ BF_4_^−^ versus Ag/0.1 M AgCl on a Bioanalytical Systems 50-W Voltammetric Analyzer. The potential of the reference electrode was +0.047 V versus SCE.
